# Complicated Crohn's-like colitis, associated with Hermansky-Pudlak syndrome, treated with Infliximab: a case report and brief review of the literature

**DOI:** 10.1186/1752-1947-1-176

**Published:** 2007-12-08

**Authors:** George Kouklakis, Eleni I Efremidou, Michael S Papageorgiou, Evdoxia Pavlidou, Konstantinos J Manolas, Nikolaos Liratzopoulos

**Affiliations:** 1 Endoscopy Unit of University General Hospital of Alexandroupolis, Democritus University of Thrace, Alexandroupolis, Greece; 2 First Department of Surgery of University General Hospital of Alexandroupolis, Democritus University of Thrace, Alexandroupolis, Greece

## Abstract

**Introduction:**

Hermansky-Pudlak syndrome (HPS) is a rare autosomal recessive inherited disorder consisting of a triad of albinism, increased bleeding tendency secondary to platelet dysfunction, and systemic complications associated with ceroid depositions within the reticuloendothelial system. HPS has been associated with gastrointestinal (GI) complications related to chronic granulomatous colitis with pathologic features suggestive of Crohn's disease. This colitis can be severe and has been reported to be poorly responsive to medical therapies including antibiotics, corticosteroids, sulfasalazine, mesalamine and azathioprine.

**Case presentation:**

We report a patient with HPS which was complicated by inflammatory bowel disease with clinical and pathologic features of Crohn's disease, refractory to antibiotics, corticosteroids and azathioprine. A trial of infliximab was attempted and repeated infusions produced a complete response.

**Conclusion:**

The occurrence of ileitis and perianal lesions and also the histopathological findings in our case suggest that HPS and Crohn's disease may truly be associated. Given this similarity and the failure of the standard medical therapy of corticosteroids and azathioprine, our patient received infliximab with marked clinical improvement.

## Introduction

Hermansky-Pudlak syndrome (HPS) is a complex syndrome with a triad of manifestations of tyrosinase-positive oculocutaneous albinism (Ty-pos OCA), bleeding diathesis resulting from platelet dysfunction, and systemic complications associated with accumulation of ceroid lipofusion. Complications of the syndrome such as renal failure, cardiomyopathy, fatal pulmonary fibrosis and granulomatous colitis have been described [1,2].

Specifically, the colitis is a unique type of inflammatory bowel disease which can be severe and even fatal, with clinical features suggestive of chronic ulcerative colitis and pathological features more closely similar to those of Crohn's disease [3]. It is still unclear whether the granulomatous colitis in HPS is because of ceroid deposition or reflects the coexistence of Crohn's disease and HPS.

An analysis of most reported cases, since Schinella et al. first reported granulomatous colitis in association with HPS (1980), suggests that the colitis of HPS is simply a reaction to the tissue deposition of ceroid [4]. Yet, there are reported cases of HPS with gastrointestinal complications related to chronic granulomatous colitis, enterocolitis, ileitis, intestinal fistulization or granulomatous perianal disease [2-5]. These observations suggest that the colitis of HPS is due to the development of classical Crohn's disease. Therefore, it is possible that treatments known to be effective for Crohn's disease would be effective for HPS-associated enterocolitis [3,4]. A review of reported cases reveals no consistent success with the standard medical treatment including sulfasalazine, mesalamine, corticosteroids and antibiotics, such metronidazole and ciprofloxacin [3]. In some cases surgical intervention is necessary, while subtotal colectomy or total proctocolectomy has been performed as a last resort [1].

We, therefore, report here the occurrence of a classical clinical and pathologic picture of Crohn's disease in a woman with HPS and our experience with infliximab in treating successfully this HPS-associated enterocolitis.

## Case presentation

A 42-yr-old muslim woman with hallmark findings of HPS presented to the University Hospital of Alexandropoulos in 2005 complaining of an 10-month history of recurrent episodes of abdominal pain worse with defecation and intermittent bloody diarrhea. She had lost 10 pounds over 6 months. According to her history, HPS was diagnosed elsewhere in 2002 [tyrosinase-positive oculocutaneous albinism (Ty-pos OCA) and normal platelet count with small quantity of dense bodies (DB), without genetic linkage analysis]. Family history was significant with a sister with albinism who had died at age 36.

Initial physical examination revealed an albino woman with whitish hair, pale and unpigmented skin and strabismus. Ocular examination showed horizontal nystagmus with reduced vision and no pigmentation of the iris. Cardiopulmonary and abdominal examinations were normal. Chest x-ray and high-resolution CT didn't show any signs of pulmonary fibrosis. Baseline laboratory values were within normal range, including a normal platelet count (401 × 10^9^/L) and no signs of hemorrhagic diathesis were observed. However, platelets were small in size (MPV 8fL). Platelet aggregation tests (PFA with collagen/ADP and collagen/epinephrine) were within normal range. Bone marrow aspiration revealed the presence of pseudo-Gaucher-like appearance histiocytes.

Differential diagnosis of the colitis included the granulomatous enterocolitis found in HPS-patients, ulcerative colitis, Crohn's disease, tuberculous (TB) colitis and other forms of inflammatory bowel disease. For the possibility of tuberculosis of the intestine, at first, a tuberculin skin test was performed, which was negative.

**Figure 1:**
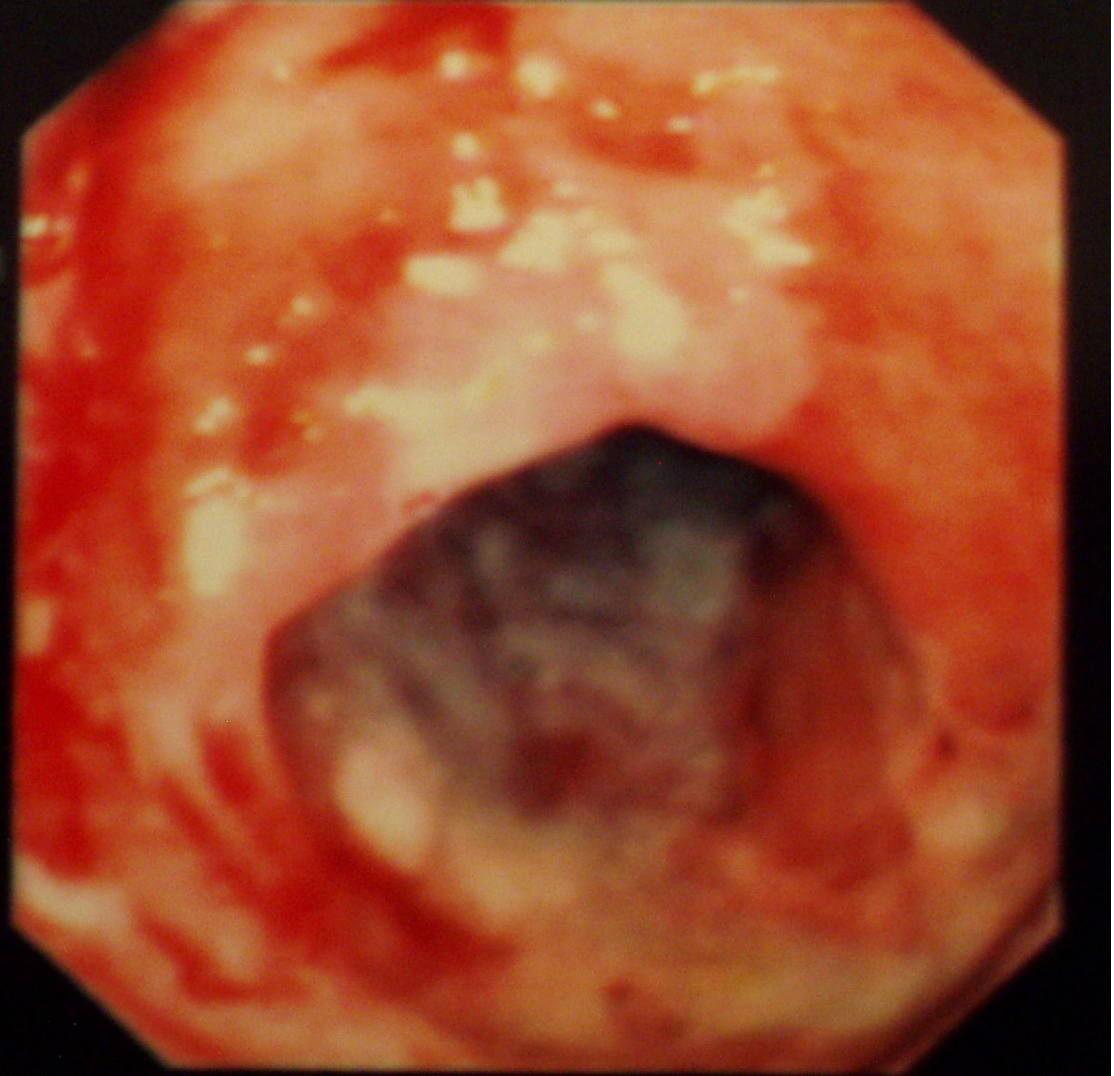
Colonoscopy of the sigmoid colon: colitis with edema, erythema and multiple ulcers.

**Figure 2:**
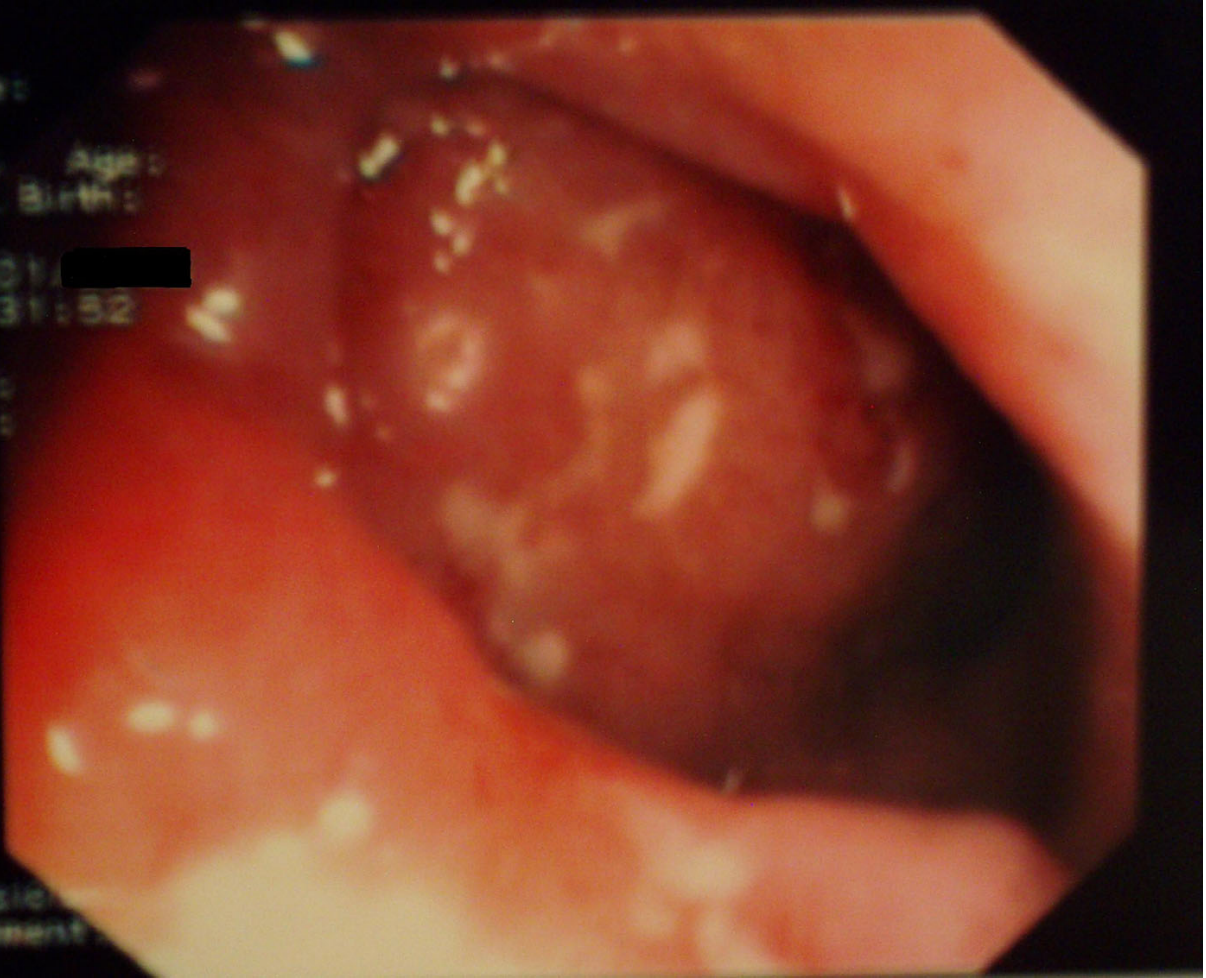
Colonoscopy of the terminal ileum: ileitis with multiple aphthoid ulcers.

Rectal examination revealed skin tags and a posterior fissure with no obvious abscess. Colonoscopy revealed severe segmental colitis with multiple ulcers in the sigmoid colon (Figure 1) and numerous deep ulcers in the terminal ileum (Figure 2), with sparing of the remainder of the colon. No active bleeding was seen. This endoscopic appearance was highly reminiscent of Crohn's disease. Upper endoscopy showed no evidence of upper GI bleeding or other pathology. Contrast enhanced CT of the abdomen showed thick-walled loops of ileum and sigmoid colon, without evidence of obstruction or perforation. Histologic findings of biopsy samples from the ileum and sigmoid showed focal chronic inflammation, irregular villous architecture and granuloma formation with no obvious ceroid deposition. All the mucosal biopsies from the ileum and different colonic segments, included those from ulcer bases, were negative for *Mycobacterium tuberculosis*.

Over the next two weeks her symptoms progressed with increased pain with defecation and bloody diarrhea. During this time, the patient was treated with parenteral nutrition, steroids and antibiotics (metronidazole and ciprofloxacin). Treatment with azathioprine (AZA) 100 mg/daily was begun with little effect. This therapy resulted in temporary improvement but two weeks later the patient's overall condition acutely worsened and she developed drainage from the perianal fistula.

Colonoscopy at that time revealed severe ileitis, multiple linear ulcers with edema and erythema in the sigmoid colon and mild proctitis.

She was started on infliximab 5 mg/Kg and azathioprine 100 mg/daily. Within 72 hours this treatment led to improvement in symptoms. The patient had soft tools without blood and only minimal pain and drainage from the fistula.

**Figure 3:**
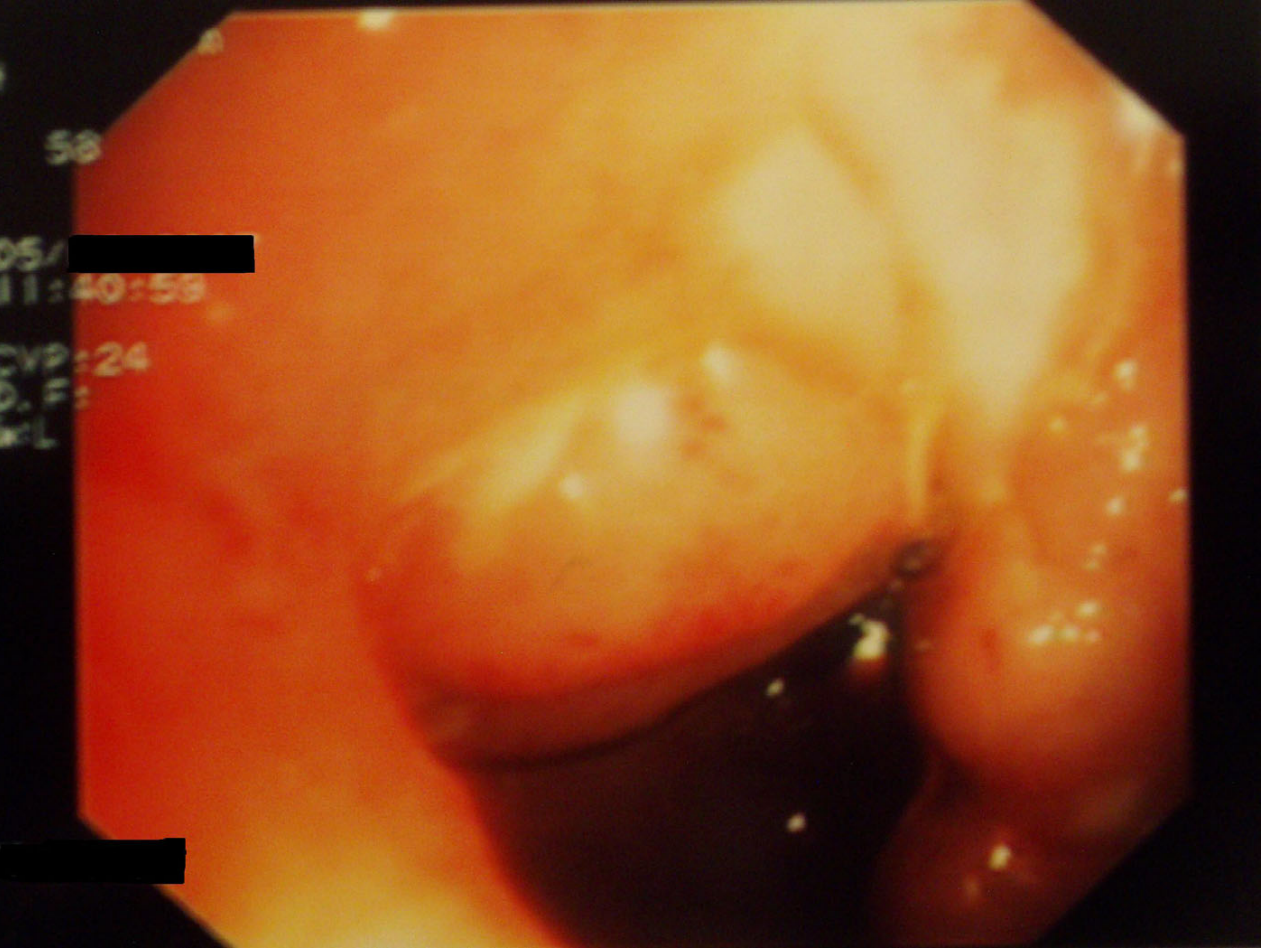
Improvement of ileitis, healing of ulcers (after 8 wk of the initial dose of infliximab).

**Figure 4:**
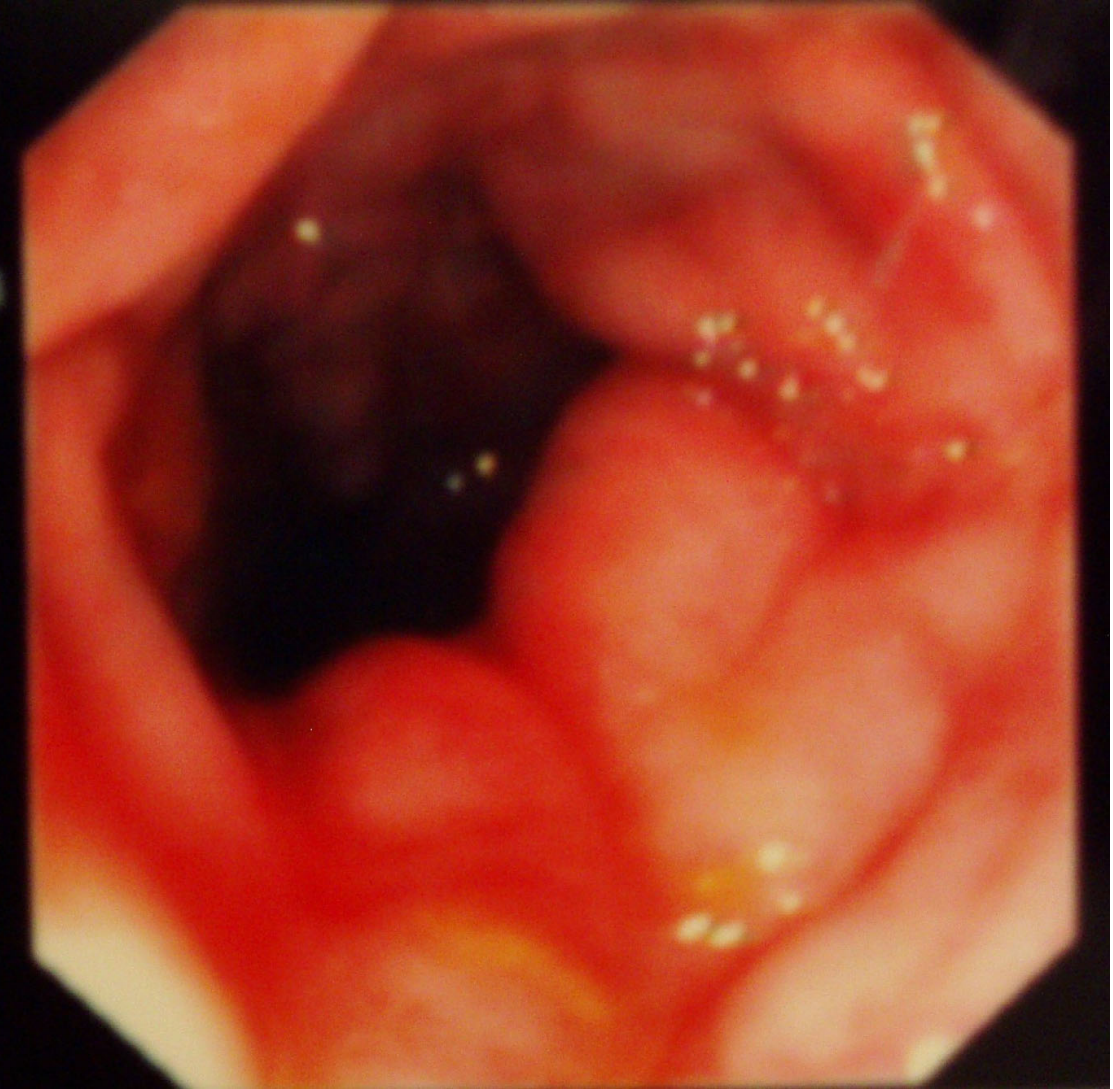
Improvement of edema and healing of linear ulcers in sigmoid colon (8 weeks after the initial dose of infliximab).

She again received infliximab (5 mg/Kg) at 2 and 6 weeks following the initial dose with marked improvement of her colitis, as seen on repeat colonoscopy, (Figures 3 and 4) and healing of the fistula.

On a maintenance regimen of AZA 100 mg/daily and a dose of infliximab 5 mg/Kg/8 wk the patient has remained well for the past 13 months.

## Discussion

Granulomatous colitis was an unrecognized complication of HPS until its first description by Schinella et al [1]. Since then, HPS-associated colitis has been reported several times. Witkop et al [6,7], Sherman et al [8] and recently Hussain et al [9], Hazzan et al [3] and Grucela et al [4] all described patients with HPS complicated by granulomatous colitis, enterocolitis and, in some cases, perianal disease.

A review of the English language medical literature identified a total of 13 patients with HPS who required surgery due to lower GI bleeding, intractable colitis or perianal disease [3]. The granulomatous colitis associated with HPS usually manifests in the first and second decades and has been described as having a clinical presentation similar to chronic ulcerative colitis and pathologic findings consistent with Crohn's disease [3,4,8].

Colonoscopy commonly reveals multiple scattered superficial and deep ulcers, with pseudopolyps in some cases, from the rectum to cecum [4]. To our knowledge small bowel inflammation has been described in only three patients [1,3].

Histologically, broad ulcers, which extend into the muscularis mucosa, brown granular pigmentation and non-necrotizing granulomas are seen [1,9,10]. Specifically it has been noted that the granulomas are not formed in relation to deposits of the ceroid-like pigment [1].

In our case, the patient was found to have segmental severe granulomatous colitis with linear ulcers in the sigmoid colon and multiple aphthoid ulcers in the terminal ileum while pathological features were almost indistinguishable from those of Crohn's disease,, including chronic inflammation, crypt architectural abnormalities and epithelioid granulomas without ceroid deposition.

In addition, our patient had perianal disease with inflammation and fistula, a complication characteristic of Crohn's disease that has been previously described by Sherman et al in 1989 [8] and by Hazzan et al in 2006 [3]. The clinical and pathologic findings described herein are most consistent with a Crohn's-like phenotype.

The pathogenesis of bowel involvement in HPS is unknown. It has been suggested that the granulomatous colitis of HPS results from the accumulation of ceroid-like pigment in intestinal macrophages, rupture of them, release of lysosomal hydrolases and resultant tissue damage [1,3,4,7-9]. An autopsy study by Sherman et al. showed deposition of ceroid-like pigment throughout the GI tract, which was often associated with lymph follicles in the absence of colitis [8]. In the case of granulomatous colitis, pigment remained sparse but was markedly increased in pericolonic lymph nodes draining areas of active colitis [8]. Regarding the absence of ceroid pigment in our case, others have noted that relatively small quantities of ceroid are deposited in the bowel and that any deposits can be very focal [1]. Previously published reports have indicated that medical treatment of granulomatous colitis associated with HPS has been unsuccessful. Given the clinical similarity of HPS colitis to Crohn's disease, the therapeutic approach to patients with HPS-associated colitis is similar to treatment for Crohn's disease, including corticosteroids, antibiotics and immunomodulators.

However, a review of the literature reveals no consistent success with the standard medical therapy used in treating Crohn's disease and in some of these cases surgical intervention was necessary [3]. Infliximab is a chimeric monoclonal antibody which has recently proven effective in patients with Crohn's disease refractory to current medical therapy and it is also used as a first line agent in severe perianal fistulizing disease. There are recently published reports of patients with HPS complicated by granulomatous colitis refractory to medical therapy with antibiotics, corticosteroids and immunomodulators but responsive to treatment with infliximab [2,4,5].

This was the finding in our patient as well; treatment with infliximab showed immediate improvement and there was a complete response to repeated infusions of infliximab. This is the second report of HPS-associated granulomatous colitis with severe Crohn's-like ileitis which dramatically responded to treatment with infliximab.

## Conclusion

It is still unclear if the granulomatous colitis associated with HPS is part of the syndrome or if it represents an independent but associated process, such as Crohn's disease. The fact is that granulomatous enterocolitis in several patients with HPS phenotypically appears like, and clinically behaves like, Crohn's disease. It is also a fact that infliximab results in a response in some of these patients.

In addition, the fact that infliximab results in a response in some of these patients suggests that HPS may be linked to Crohn's disease, or at least that TNF-a plays a pivotal role in both types of colitis.

It should be noted that treatment success with infliximab is still based on limited experience and only in patients with HPS-associated Crohn's-like enterocolitis and it should not be generalized as the optimal therapeutic approach to all patients with granulomatous colitis complicating HPS. More research is needed to determine the cause of the granulomatous enterocolitis of HPS and also possible common genetic linkages with Crohn's disease.

## Competing interests

The author(s) declare that they have no competing interests.

## Authors' contributions

GK conceived of the study and participated in its design and coordination. EIE participated in the design of the study, the acquisition and interpretation of data and drafted the manuscript. MSP participated in the sequence alignment and reviewed the literature. EP participated in the sequence alignment and drafting the manuscript. KJM participated in the conception of the study, revised it for intellectual content and gave final approval of the version to be published. NL participated in the design of the study and coordination and helped to revise the manuscript. All authors, contributed intellectual content, have read and approved the final manuscript.

## Consent

The authors state that written informed patient consent was obtained for publication.
